# W Prime: Evidence-Based Proposal for a New Predictor of Gait Speed in Older Women

**DOI:** 10.3390/muscles2030022

**Published:** 2023-08-24

**Authors:** Gersiel Nascimento de Oliveira Júnior, Jairo de Freitas Rodrigues de Sousa, Marcelo Augusto da Silva Carneiro, Fernanda Maria Martins, Samarita Beraldo Santagnello, Rosekeila Simões Nomelini, Cláudio de Oliveira Assumpção, Markus Vinícius Campos Souza, Fábio Lera Orsatti

**Affiliations:** 1 Applied Physiology, Nutrition and Exercise Research Group (PhyNER), Exercise Biology Research Laboratory (BioEx), Institute of Health Science, Federal University of Triangulo Mineiro (UFTM), Uberaba 38025-180, MG, Brazilclaudio.assumpcao@uftm.edu.br (C.d.O.A.); markusviniciuscampos@gmail.com (M.V.C.S.); 2 Metabolism, Nutrition, and Exercise Laboratory, Physical Education and Sport Center, State University of Londrina, Londrina 86057-970, PR, Brazil; 3 Gynecology and Obstetrics Program, Oncology Research Institute (IPON), Federal University of Triangulo Mineiro (UFTM), Uberaba 38025-180, MG, Brazil; 4 Department of Sport Sciences, Health Science Institute, Federal University of Triangulo Mineiro (UFTM), Uberaba 38025-180, MG, Brazil

**Keywords:** aging, critical torque, fatigue, physical function

## Abstract

Background: The hyperbolic torque-duration curve depicts critical torque (CT) and W prime (W′), with the curve’s asymptote representing CT as the boundary between heavy- and severe-intensity domains. W′, the curvature constant, indicates cumulative work beyond CT. This study investigated age-related reductions in W′, CT, and gait speed, and whether W′ and CT predict gait speed independently of muscle torque. Methods: three groups (adults, middle-aged, older) totaling 131 women were studied. W′ and CT were determined using 60 maximal isometric voluntary contractions of knee extensors. The fast gait speed was calculated in walking tests at 10 m, 400 m, and six minutes (6 MWT). Results: gait speed decreased (*p* < 0.05) with age, as did W′ and CT. Both W′ and CT correlated positively with gait speed at different distances (10 m, 400 m, 6 MWT). Adjusted for maximum muscle torque, only W′ maintained a positive association (*p* < 0.05) with all gait speed tests (10 m: β = 0.201, SE = 0.086; 400 m: β = 0.262, SE = 0.085; 6 MWT: β = 0.187, SE = 0.086). Conclusions: aging led to declines in W′, CT, and gait speed. W′, not CT, remained a significant predictor of gait speed, indicating its importance for older women’s mobility.

## 1. Introduction

Women spend about one-third of their lives in the postmenopausal period (typically after the age of 50). The menopausal transition, which occurs between the ages of 45 and 55, is marked by a significant decrease in muscle torque and gait speed [1,2]. Low gait speed is directly associated with increased risks of falling, disability, functional dependence [3], and mortality in older adults [4,5]. This raises healthcare expenses for community-dwelling older persons [6,7], with older women bearing a disproportionate share of the burden [8].

The decline in muscle function, specifically maximal muscle torque due to aging, is recognized as a crucial influence on the reduced gait speed seen in older individuals [9,10,11]. Fatigability, the rapid decrease in maximal muscle torque during contractile activity, has emerged as a significant contributor to this decline in gait speed among older individuals [12]. In contrast to younger counterparts, older individuals require greater effort—approaching their maximum muscle strength—to achieve higher gait speeds [13,14]. This heightened effort can lead to muscle fatigue, limiting the ability of older individuals to sustain higher muscle torque during walking tests [15,16,17]. While women generally experience lower fatigability than men, this discrepancy appears to be affected by women’s hormonal state [18], and the effect diminishes after the transition to menopause. Therefore, assessing the decline in muscle torque resulting from contractile activity (fatigability) may more accurately reflect the performance of fast walking tests in older individuals compared to measuring maximal muscle strength [16].

Although a comprehensive understanding of the mechanisms driving the age-related escalation in muscle fatigability remains incomplete, several contributing factors have been identified. These encompass the waning oxidative capacity of muscles associated with the aging process [19], an augmented accumulation of metabolites within muscle tissue [20], and a decline in contractile economy—marked by an age-related elevation in the ATP cost during muscle contractions [21]. Collectively, these factors synergize to underpin the diminishing muscle function and declining physical performance characteristic of advancing age [22]. Numerous studies have indicated that the primary source of increased fatigability during exercise is located primarily within the muscle tissue itself, as opposed to originating from neural influences [12,23,24].

Various methodologies have been employed to assess fatigability [12,15,16,17,23,24,25,26]. Among these methods, a practical framework for investigating fatigability thresholds and physical performance is the relationship between muscle torque (or muscle power) and time-to-exhaustion, as discussed in reference [12]. This relationship is visually represented by a hyperbolic curve, yielding two distinct parameters: critical torque (CT) and W prime (W′), as elaborated upon in references [12,23,24,27]. The point at which this curve levels off (asymptote) signifies the CT, demarcating the boundary between heavy- and severe-intensity domains. This demarcation is crucial, signifying the boundary between manageable and unsustainable exercise efforts [12,23]. Furthermore, the curvature constant of this hyperbolic relationship, quantified as W′, represents the cumulative work or impulse that can be executed beyond the critical torque [12,24]. Depletion of W′ during any activity performed within the severe-intensity domain coincides with task failure and the accumulation of fatigue-related metabolites. This premise suggests that W′ may be influenced by a specific threshold of fatigue during exercises within severe-intensity domains [12,24]. Consequently, W′ serves as a constant indicative of fatigability. It functions as a marker of muscle capacity to sustain exertion above the CT during exercise efforts [12].

Both W prime (W′) and critical torque (CT) have shown reductions in older adults when compared to their younger counterparts [12,25,26]. As a result, it has been theorized that the age-related decline in W′ and CT could potentially curtail the functional capabilities of older individuals, hampering their performance in everyday tasks [12,28]. However, there exists a dearth of information concerning the role played by W′ and CT within the context of aging. Indeed, concrete evidence pertaining to the contribution of W′ and CT to the decline in gait speed performance during the aging process is notably lacking.

Therefore, the current study embarked on two primary objectives. Firstly, the investigation aimed to discern whether reductions in W′, CT, and walking speed are evident in middle-aged women (around menopause) or solely in older women when compared to their younger counterparts. Secondly, the study aimed to unravel the potential of W′ and CT as predictive indicators for gait speed, independent of maximal muscle torque.

## 2. Results

All 131 participants completed the study. Medication intake, demographics, and anthropometric characteristics of all groups are detailed in Table 1.

Total fat was higher in the middle-aged group compared to other groups, and even higher in the older group than in the young group (*p* < 0.05). Fat (%) was lower in the young group compared to the other groups (*p* < 0.05), with no significant difference between the middle-aged and older groups (*p* > 0.05) (Table 2).

TLM, maximal muscular torque, 10 m, 400 m, and 6 MWT, progressively decreased (*p* < 0.05) from the young group to the older group (Table 2). These parameters decreased as follows: TLM by 13.0%, maximal muscular torque by 35.8%, 10 m by 20.8%, 400 m by 19.0%, and 6 MWT by 19.0% (corresponding to annual reductions of 0.5%, 0.8%, 0.5%, 0.4%, and 0.4%, respectively) from the young group to the older group.

**Figure 1:**
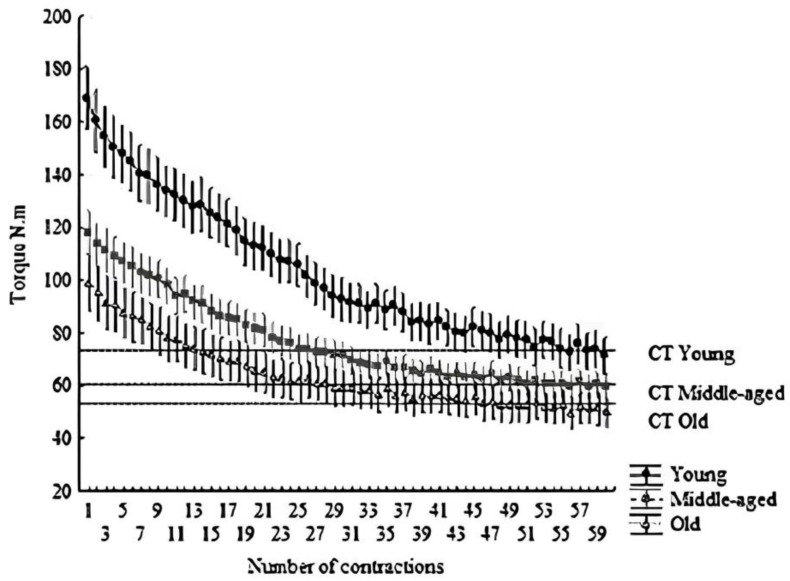
Hyperbolic relationship between torque and time. The dashed line indicates the CT. All points above the line form the area under the curve, indicating W′. Mean ± SD of each contraction (total of 60 contractions, 300 s).

**Figure 2:**
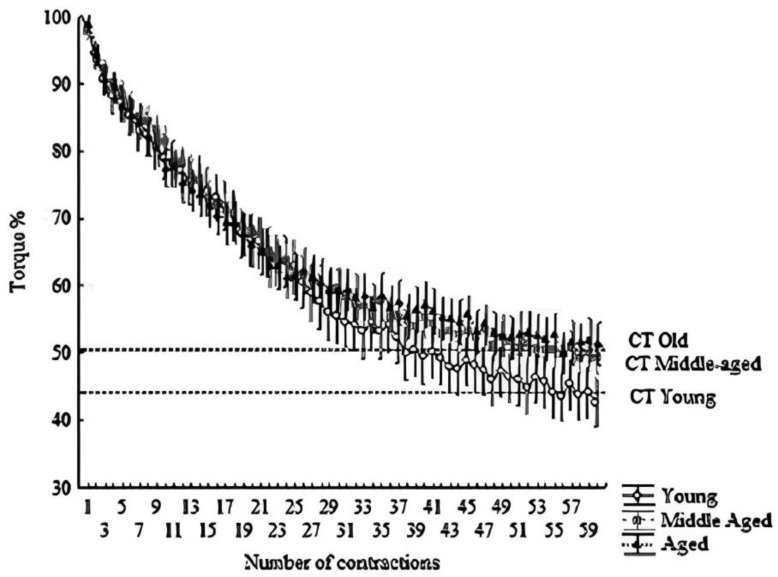
Hyperbolic relationship between relative torque (%) and time. The dashed line indicates the CT. All points above the line form the area under the curve, indicating W′. Means ± SD of each contraction (total of 60 contractions; 300 s).

Similarly, W′, W′%, W′/maximal muscular torque, CT, CT%, and CT/maximal muscular torque decreased progressively (*p* < 0.05) from the young group to the older group. However, no significant differences (*p* > 0.05) were observed between the middle-aged and older groups for W′%, CT, CT%, and CT/maximal muscular torque. The W′ decreased by 54.9% (~1.3% per year), while CT decreased by 26.5% (~0.6% per year) from the young group to the older group (refer to Figure 1 and Figure 2).

W′, maximal muscular torque, and TLM showed positive associations with gait speed at 10 m, 400 m, and 6 MWT (*p* < 0.05). However, after adjusting for maximal muscular torque, only W′ (W′/maximal muscular torque) and W′% (the percentage of W′ relative to maximal muscle torque) remained positively correlated with gait speed at 10 m, 400 m, and 6 MWT (*p* < 0.05). CT/maximal muscular torque exhibited a negative association with gait speed at 10 m and 400 m (*p* < 0.05), while no significant association (*p* < 0.05) was found between CT% and gait speed (Table 3).

## 3. Discussion

There is limited evidence concerning the relationship between W′ and CT in the context of aging. Specifically, the role of W′ and CT in the age-related decline of gait speed lacks comprehensive investigation. Consequently, our study aimed to address two key objectives: firstly, to explore potential reductions in W′ and CT, along with their correlation to gait speed, in older women compared to their younger counterparts; secondly, to assess whether W′ and CT independently predict gait speed, even when accounting for maximal muscle torque. Our primary findings demonstrate that, firstly, W′, CT, and gait speed experience a progressive decline with advancing age. This decline becomes evident even among middle-aged women. Secondly, the decrement in gait speed can be partially attributed to the reduction in both W′ and CT. However, following adjustments for maximal muscle torque, only W′ maintains its status as a predictor of gait speed (refer to Table 3). Consequently, our study makes a significant contribution to the existing literature by supporting the hypothesis that the decline in W′ (reflecting increased fatigability), but not in CT, during the aging process may constrain the ability of older adults to achieve adequate gait speed. Moreover, our findings suggest that W′ holds potential as a therapeutic avenue for enhancing or preventing low gait speed in older adults. Therefore, the confirmation of this hypothesis would require randomized clinical trials. This is of paramount importance due to the strong association between low gait speed and elevated risks of falling, disability, functional dependence, and mortality among older individuals. Thus, our study underscores the significance of W′ as a potential factor influencing gait speed decline in older adults. The implications of our findings open the door to potential therapeutic interventions aimed at addressing gait-related issues in this population. Further research, particularly randomized clinical trials, will be crucial in establishing the efficacy of such interventions and improving the overall well-being and mobility of older individuals [4,5].

Our results indicate lower W′ and CT values in the older group compared to the younger group, suggesting a decline in both variables with aging. This finding aligns with Neder et al. [25] and Overend et al. [26], who also reported decreased W′ and CT values in older adults compared to their younger counterparts. Notably, while these studies demonstrated a similar decline in W′ and CT from young to older adults, our results indicate a more pronounced reduction in W′ than in CT (W′ = 54.9%, ~1.3% per year vs. CT = 26.5%, ~0.6% per year). However, there is a scarcity of data on the aging-related decline of W′ and CT, leading to a limited understanding of the temporal pattern of reduction in these variables with age. The variation in measurement methods (cycling vs. isometric) and sample characteristics (men vs. women; active vs. sedentary) between our study and others [25,26] further complicates direct comparisons.

While a direct comparison between our results and previous studies proved elusive, several factors likely contribute to the intolerance of severe-intensity exercise in older adults. These factors encompass reduced muscle mass, primarily attributed to type II muscle fiber atrophy [29], compromised neural control over skeletal muscle contractile function [21], diminished anaerobic metabolism capacity for energy production during intense contractions [30], decreased perfusion of skeletal muscle [31,32], and inflammation [33]. Moreover, aging’s impact on muscle mass and function can be expedited during the menopausal transition [34,35,36]. This diminished capacity for high-intensity exercise, coupled with declining muscle strength, prompts an increased reliance on oxidative sources to sustain ATP production during daily activities among older individuals. Indeed, our observations reveal a significantly higher CT relative to maximal torque (CT%) in older adults compared to their younger counterparts (Table 2 and Figure 1 and Figure 2). Similarly, Overend et al. [26] demonstrated a markedly greater critical power relative to maximal power output in older adults than in the young. Moreover, these findings find resonance in earlier meta-analyses [34,37], indicating that older adults may exhibit lower fatigue susceptibility (i.e., better maintenance of submaximal force) compared to younger adults, especially in protocols involving low intensities relative to maximal performance. Collectively, these insights suggest that muscle endurance-related parameters may exhibit a more pronounced preservation compared to muscle maximal capacity in the context of aging. Consequently, W′ might offer greater sensitivity in detecting age-related changes in muscle function than CT.

Previous research has established that maximal muscle strength serves as a reliable indicator of gait speed [9,10,11]. However, the heightened effort demanded for brisk walking tests among older adults [13,14] can induce muscle fatigue, known as fatigability. This fatigability might curtail the ability to sustain elevated muscle torque during walking tests over a specific duration [15,16,17]. Notably, our findings underscore a correlation between gait speed and both W′ and CT. Furthermore, the predictive potency (beta value) of W′ closely parallels that of maximal muscle torque concerning gait speed within our study’s cohort (Table 3). This concurs, at least partially, with earlier investigations suggesting that the capability to sustain heightened muscle exertion corresponds to gait speed [12,28]. Furthermore, our results reveal that after adjusting W′ and CT in relation to maximal muscle torque, only W′ (W′/torque and W′%) maintains a positive correlation with gait speed (Table 2). This implies that the ability to sustain heightened muscle effort beyond the critical threshold (i.e., W′) serves as a predictive factor for gait speed performance, irrespective of maximal muscle strength, among older adults. Thus, our findings propose that evaluating W′ offers an additional predictor of gait speed.

Given that W′ serves as a predictive factor for gait speed, enhancing W′ (i.e., reducing fatigability) holds promise as a potential therapeutic approach to enhance or prevent reduced gait speed in older adults. In a recently published perspective, Denadai and Greco proposed that an elevated W′ might underlie changes in physical capacity following resistance training [28]. Specifically, resistance training involving higher loads could potentially lead to enhancements in muscle buffer capacity (i.e., W′) [35], heightened rates of ATP synthesis from anaerobic glycolysis, and increased expression of fast myosin heavy chain, along with improved PCr resynthesis [36]. These enhancements, resulting from resistance training with higher loads, could ultimately augment exercise tolerance (W′) [38], enabling older adults to sustain superior gait speed performance. Consequently, we emphasize the importance of engaging in resistance training with higher loads to optimize gait speed. It is important to note, however, that further studies are required to elucidate the validity of this possibility.

This study bears acknowledgment of three inherent limitations. Firstly, it did not encompass the measurement of fear of falling, a factor that can adversely impact gait speed in older adults, regardless of their physical capability to achieve greater speeds. Secondly, a methodological constraint arises from the study’s cross-sectional design, which precludes the establishment of causal relationships. Notably, protracted longitudinal investigations spanning over three decades or more are intricate to execute and economically burdensome. Hence, the cost-effectiveness and efficiency of cross-sectional studies remain essential avenues for uncovering fresh insights, potentially compensating for the absence of longitudinal data. Furthermore, the study yielded substantial effect size and power, bolstering the plausibility of generalizable outcomes (external validity). Secondly, it’s imperative to acknowledge the study’s confinement to a specific demographic, namely women, with the older cohort predominantly composed of individuals aged 60 to 70. This limited scope narrows the representation of “older women” and, as such, the outcomes may not be extrapolated to women aged >70. Furthermore, given women’s documented resilience against fatigue in contrast to men [39], the observed effects may not uniformly apply across varying contexts. Thus, future investigations should encompass a broader demographic, encompassing women above 60 years and potentially diversifying the sample to include men. Lastly, it’s worth noting that while other muscles/movements like hip extension and ankle plantar flexion contribute more substantially to gait propulsion on level surfaces, this study opted for knee extensors due to the development and validation of the torque–time relationship protocol (W′ and CT) in knee extensors [27]. Knee extensors exhibit a higher proportion of type II fibers relative to other muscles [40,41], rendering them significant indicators of age-linked functional deterioration and fatigability [42]. The fatigability of knee extensors has been linked to age-associated variations in functional performance [17,18,20], thereby justifying their selection for this study.

## 4. Materials and Methods

### 4.1. Study Design

This cross-sectional study aimed to compare W′, CT, and gait speed across distinct age groups (young, middle-aged, and older women). Furthermore, it sought to determine whether W′ and CT serve as predictors of gait speed, independent of maximal muscle torque. The study enlisted women aged between 18 and 85 years as participants. Over a span of four days, participants visited the laboratory at consistent times (08:00 and 10:00 a.m.) to mitigate the influence of diurnal biological fluctuations. The assessments were conducted in the subsequent sequence: on the first day, anthropometric and body composition measurements were taken; the second day involved maximal isometric voluntary contractions, CT, and W′ evaluations; on the third day, gait speed assessments (10-m, 400-m, and 6-min walk tests) were performed; and on the fourth day, all gait speed tests were re-administered for validation. Furthermore, two different examiners conducted the assessments: Examiner A oversaw muscle strength, CT, and W′ measurements, while Examiner B supervised gait speed evaluations. The examiners remained unaware of each other’s observations. The same examiner conducted both the initial assessments and retests. The gathered data were collated in a spreadsheet for subsequent statistical analysis. Following the evaluation of all volunteering participants, the women were categorized into distinct age groups: younger adults (18–39 years old), middle-aged adults (40–59 years old), and older adults (>60 years old).

### 4.2. Participants

The a priori sample size for the F-test was determined using G*Power (v. 3.0.10, Düsseldorf, Germany). Drawing on W′ outcomes from Neder et al.’s study [25] (comparing young and older adults; Cohen’s d = 1.5), we established an alpha level of 0.05, a power of 80%, and a substantial effect size (eta = 0.14). Consequently, a minimum of 66 participants (22 participants per group) was deemed necessary for this investigation. However, the study garnered participation from 131 women, exceeding the anticipated number.

Recruitment took place through invitations extended at local community centers near the university, both in person and via social media channels. Prospective volunteers underwent screening involving an anamnesis comprising queries regarding demographic particulars, current health conditions, and the consumption of alcohol or tobacco. The final sample encompassed 131 women who met the specified inclusion criteria, which encompassed: refraining from hormone therapy or phytoestrogen use; maintaining controlled blood pressure and glycemic levels; absence of myopathies, arthropathies, and neuropathies; no history of muscular, thromboembolic, or gastrointestinal disorders; lack of cardiovascular or infectious ailments; non-consumers of alcohol (complete abstinence from alcohol intake in their diet); and non-smokers.

The volunteers were assigned to three distinct groups: (1) individuals aged 18–39 (young; n = 29); (2) those aged 40–59 (middle-aged; n = 57); and (3) individuals aged ≥60 (older; n = 45). The older group comprised 32 women aged between 60 and 69, 11 women aged between 70 and 79, and 2 women aged between 80 and 89.

The study obtained approval from the local ethics committee, and all participants provided their informed consent by signing a designated form before engaging in the study. All procedures adhered to the Helsinki Declaration, revised in 2008.

### 4.3. Anthropometric and Body Composition Assessments

Measurement of body weight and height was carried out utilizing a digital scale (Lider^®^, Araçatuba, Brazil) and a stadiometer firmly affixed to the scale. To ascertain body composition, encompassing total body fat mass, body fat percentage (%), and lean mass of the right thigh (TLM), dual-energy X-ray absorptiometry (Lunar iDXA; GE, Madison, WI, USA) was employed. Quantification was executed via Encore Software, version 14.10. TLM of the right thigh was precisely demarcated, with the distal boundary set at the tibiofemoral joint and the proximal boundary established as a diagonal bifurcation through the femoral neck [43]. DXA assessments were undertaken following an 8–10 h fasting period. In order to standardize body hydration levels, participants were instructed to ingest 2 L of water the day preceding the DXA assessment. Volunteers were attired in clothing devoid of any metal objects and were directed to void their bladders prior to the assessment. All evaluations were administered by the same proficient examiner.

The TLM quantified through DXA has demonstrated a robust correlation (R2 = 0.88; *p* < 0.001) with TLM quantified via magnetic resonance imaging [44]. Furthermore, DXA is widely acknowledged as a reference standard for lean mass measurement [44].

### 4.4. Gait Speed Assessment

Prior to the gait speed evaluations, all participants engaged in a warm-up phase by walking for five minutes at their customary pace. The calculation of fast gait speed (m/s) was executed during walking tests conducted at distances of 10 m (10 m) [45], 400 m (400 m), and within a span of six minutes (6 MWT) [46,47].

All walking tests transpired indoors on a level surface within a sports court measuring 32 m + 19.5 m + 32 m + 19.5 m in length. A prominently colored tape was employed to demarcate the commencement and conclusion of each 103 m lap on the floor. To ensure maximal effort, participants were motivated (e.g., through verbal encouragement such as “you are doing great” and “walk as fast as you can”) to maintain their swiftest pace without interruptions during all testing sessions. A qualified professional supervised all walking tests to ensure consistency.

Each walking test was performed twice, with a two-day separation (48 h) between test and retest sessions. The optimal gait speed, whether from the initial test or retest, was recorded for analysis. The reliability of test-retest outcomes was quantified using the intraclass correlation coefficient (ICC), accompanied by a 95% confidence interval (CI 95%). Impressively, all three walking tests (10 m, 400 m, and 6 MWT) exhibited exceptional test-retest reliability: ICC = 0.940, CI 95% = (0.904–0.961) for 10 m; ICC = 0.929, CI 95% = (0.897–0.951) for 400 m; and ICC = 0.978, CI 95% = (0.965–0.986) for 6 MWT.

### 4.5. Maximal Isometric Voluntary Contraction Assessment

Maximal isometric voluntary contractions (MIVC) were conducted within a controlled laboratory environment maintained at a climate of 21–25 °C. The right leg was affixed to the dynamometer’s lever arm, positioned just above the medial malleolus, with the ankle joint unbound to static fixation. Participants assumed a seated posture and were securely fastened to the testing chair. The hip and knee joints were set at angles of 100° and 70°, respectively. Trunk mobility was confined using cross-shoulder harnesses and an abdomen belt. Hands were situated atop the cross-shoulder harnesses for support. To initiate, participants underwent a 2 min warm-up comprising 24 contractions, each spanning 3 s of contraction followed by 2 s of rest, with a submaximal effort described as comfortable.

Subsequently, participants were instructed to execute the MIVC with utmost swiftness and force. Three MIVC measurements were executed, with a three-minute intermission between each iteration. The sequence of contractions and rest periods was overseen by a metronome, synchronized with verbal cues of “go” and “stop”. The force signal (measured in kilograms) was meticulously captured (2000 Hz) using a load cell and MioGraph software (Miotec^®^, Brazil). Torque was computed by multiplying the MIVC value by the leg length, representing the distance spanning the medial malleolus to the knee intra-articular space. Exceptional reliability was observed in MIVC measurements [ICC = 0.974, CI 95% = 0.963–0.982].

### 4.6. CT and W′ Assessments

The CT and W′ assessments were administered following a 5-min rest interval subsequent to the last maximal isometric voluntary contraction (MIVC). Utilizing the same apparatus employed for MIVC, participants embarked on the CT and W′ evaluations. A regimen of 60 MIVCs, involving 3 s of contraction accompanied by 2 s of rest, was executed over a span of 5 min (refer to Figure 1) [27]. Participants were deliberately kept unaware of the test’s time duration or the total number of contractions. For each contraction, the muscle strength attained during the contraction plateau (ranging from 2 to 2.5 s) was recorded, with the ensuing average being documented. The CT was defined as the mean of the terminal six contractions within the sequence of 60 MIVCs [27]. Meanwhile, W′ encompassed all exertion surpassing the CT threshold. Employing GraphPad Prism software (version 5.0, GraphPad Software Inc., San Diego, CA, USA), the area under the curve (force vs. time) was computed, with CT serving as the delineating threshold. Additionally, W% and CT% were computed for each participant, utilizing the highest value of MIVC (maximal muscle torque) as the benchmark of 100% (see Figure 2).

For the assessment of test–retest reliability concerning W′ and CT, a subset of the sample was chosen (n = 54; (young; n = 11; middle-aged, n = 23; and old, n = 20)). Remarkably, both W′ and CT demonstrated exceptional test-retest reliability (ICC = 0.955, CI 95% = (0.919–0.974) and ICC = 0.976, CI 95% = (0.955–0.986)) respectively. Furthermore, to verify the representation of a critical threshold by the CT values acquired in our study, a distinct portion of the sample (n = 75) was chosen for the execution of two novel tests: one conducted at 10% above and the other at 10% below their critical torque. In the instance of testing at 10% below CT (54.5 Nm ± 17.7 Nm), all participants engaged in an intermittent protocol involving 3 s of contraction followed by 2 s of rest, sustained over a span of 30 min (up to the determined maximal duration). Conversely, at 10% above CT (66.6 Nm ± 17.7 Nm), volunteers executed an intermittent protocol until the point where they were unable to reach the designated value (at 10% above CT), thus concluding the test. The duration of task failure was found to be 8.6 ± 5.8 min. Notably, this duration exhibited a positive correlation with W′ (r = 0.7).

### 4.7. Statistical Analysis

Data distribution was assessed using the Shapiro–Wilk test. Group comparisons were conducted using one-way ANOVA, followed by post-hoc analysis (Newman–Keuls). For evaluating test-retest reliability, the intraclass correlation coefficient was employed. Simple linear regression analyses were performed to examine the influence of predictor variables (TLM, W′, maximal muscular torque, CT, W′%, W′/maximal muscular torque, CT%, and CT/maximal muscular torque) on gait speed (10 m, 400 m, and 6 MWT). The results are presented as means along with a confidence interval (CI 95%), beta (β) values, standard error of β (SE), and determination coefficient (R²). The significance level was set at 5%.

## 5. Conclusions

Our findings underscore a decline in W′, CT, and gait speed (both short and extended tests) with aging among women. Both W′ and CT exhibit associations with gait speed performance. Notably, W′, in contrast to CT, retains its association with gait speed even when accounting for torque. These observations imply that the capacity to sustain heightened muscular effort beyond the critical threshold (W′ or fatigability) holds significance as a predictor of gait speed in older women. Consequently, future investigations are warranted to elucidate whether enhancing muscle fatigability (W′) could serve as a potential therapeutic approach for enhancing or preventing low gait speed in older women.

## Figures and Tables

**Table 1:** Demographic, anthropometric characterization of the young, middle-aged, and older groups.

Variable	Young (n = 29) Mean (CI 95%)	Middle-Aged (n = 57) Mean (CI 95%)	Older (n = 45) Mean (CI 95%)	*p*
Age (years)	23.8 (21.8–25.7)	52.6 * (51.3–54.0)	66.6 *^,‡^ (65.2–68.3)	<0.001
Time of menopause (years)	0.0 (0.0–0.0)	6.1 * (4.6–7.6)	16.2 *^,‡^ (14.6–17.9)	<0.001
Height (m)	1.65 (1.63–1.68)	1.60 * (1.59–1.62)	1.57 *^,‡^ (1.56–1.59)	<0.001
Body weight (kg)	61.0 (56.8–65.2)	71.3 * (68.2–74.3)	64.0 ^‡^ (60.8–67.3)	<0.001
Medication intake				
Antihyperglycemic	0	1	6	
Antihypertensive	0	16	13	
Antiasthmatic	0	0	2	
Antidepressives	0	9	8	
Antianxiolytics	0	2	1	
Antihypercholesterolemia	0	8	15	
Antiulcer	0	2	0	
Calcium	0	2	2	
Thyroxin therapy	0	4	12	

Notes. CI = confidence interval; * = differences for Young (*p* < 0.05); ^‡^ = differences for Middle-aged (*p* < 0.05).

**Table 2:** Comparison of body composition, W′, CT, maximal torque, and gait speed tests between the groups.

Variable	Young (n = 29) Mean (CI 95%)	Middle-Aged (n = 57) Mean (CI 95%)	Older (n = 45) Mean (CI 95%)	*p*	Effect Size	Observed Power
TLM (kg)	5.4 (5.1–5.7)	4.7 * (4.5–4.9)	4.3 *^,‡^ (4.1–4.6)	<0.001	0.19	1.00
Total fat (kg)	18.8 (15.9–21.6)	30.4 * (28.3–32.4)	25.7 *^,‡^ (23.5–28.0)	<0.001	0.25	1.00
Fat (%)	30.4 (28.4–32.5)	41.7 * (40.2–43.2)	39.9 * (38.3–41.5)	<0.001	0.39	1.00
Maximal torque (N.m)	184.4 (170.7–198.0)	133.4 * (123.5–143.2)	118.4 *^,‡^ (107.7–129.2)	<0.001	0.31	1.00
W′ (N.m.s)	8880.6 (8115.4–9645.8)	5320.6 * (4769.9–5871.2)	4004.3 *^,‡^ (3439.2–4641.4)	<0.001	0.43	1.00
W′ (%)	5210.8 (4784.3–5637.3)	4332.1 * (4025.2–4639.0)	3953.8 * (3618.8–4288.8)	<0.001	0.13	0.98
W′/ maximal torque (N.m)	47.8 (43.9–51.8)	40.2 * (37.4–43.0)	34.4 *^,‡^ (31.3–37.5)	<0.001	0.16	1.00
CT (N.m)	73.7 (66.6–80.7)	60.3 * (55.3–65.4)	54.2 * (48.6–59.7)	<0.001	0.13	0.98
CT (%)	44.0 (40.5–47.4)	50.8 * (48.3–53.3)	50.3 * (47.6–53.0)	0.005	0.08	0.85
CT/ maximal torque (N.m)	0.4 (0.4–0.4)	0.5 * (0.4–0.5)	0.5 * (0.4–0.5)	0.032	0.05	0.65
10 m (m/s)	2.4 (2.3–2.5)	2.0 * (1.9–2.1)	1.9 *^,‡^ (1.8–2.0)	<0.001	0.38	1.00
400 m (m/s)	2.1 (2.0–2.2)	1.8 * (1.8–1.9)	1.7 *^,‡^ (1.7–1.8)	<0.001	0.41	1.00
6 MWT (m/s)	2.1 (2.0–2.1)	1.8 * (1.8–1.8)	1.7 *^,‡^ (1.6–1.7)	<0.001	0.41	1.00
TLM (kg)	5.4 (5.1–5.7)	4.7 * (4.5–4.9)	4.3 *^,‡^ (4.1–4.6)	<0.001	0.19	1.00

Notes. CI = confidence interval; * = differences for Young (*p* < 0.05); ^‡^ = differences for Middle-aged (*p* < 0.05). TLM = thigh lean mass; CT = critical torque; W′ = work above critical torque; 10-m = 10 m; 400-m = 400 m; 6 MWT = 6-min walk test.

**Table 3:** Regression analysis between gait speed (10 m, 400 m, and 6 MWT) and W′, CT, maximal torque, and TLM.

Variable	10 m				400 m				6 MWT			
	Β	SE β	R²	*p*	β	SE β	R²	*p*	β	SE β	R²	*p*
W′	0.484	0.077	0.23	<0.001	0.534	0.074	0.28	<0.001	0.504	0.076	0.25	<0.001
CT	0.325	0.083	0.10	<0.001	0.387	0.081	0.14	<0.001	0.451	0.078	0.20	<0.001
Maximal torque	0.544	0.074	0.29	<0.001	0.548	0.073	0.30	<0.001	0.566	0.070	0.32	<0.001
TLM	0.394	0.081	0.15	<0.001	0.321	0.083	0.10	<0.001	0.345	0.08	0.11	<0.001
W′%	0.233	0.086	0.05	0.007	0.269	0.084	0.07	0.002	0.188	0.086	0.03	0.032
W′/Maximal Torque	0.201	0.086	0.04	0.021	0.262	0.085	0.07	0.002	0.187	0.086	0.03	0.032
CT%	−0.005	0.009	0.00	0.955	−0.096	0.087	0.01	0.274	−0.051	0.088	0.00	0.564
CT/Maximal Torque	−0.248	0.085	0.06	0.004	−0.179	0.086	0.03	0.039	−0.110	0.088	0.01	0.211

Notes. 10 m = 10 m; 400 m = 400 m; 6 MWT = 6-min walk test; W′ work above critical torque; CT = critical torque; TLM = thigh lean mass.

## Data Availability

The authors can provide the data when required.
